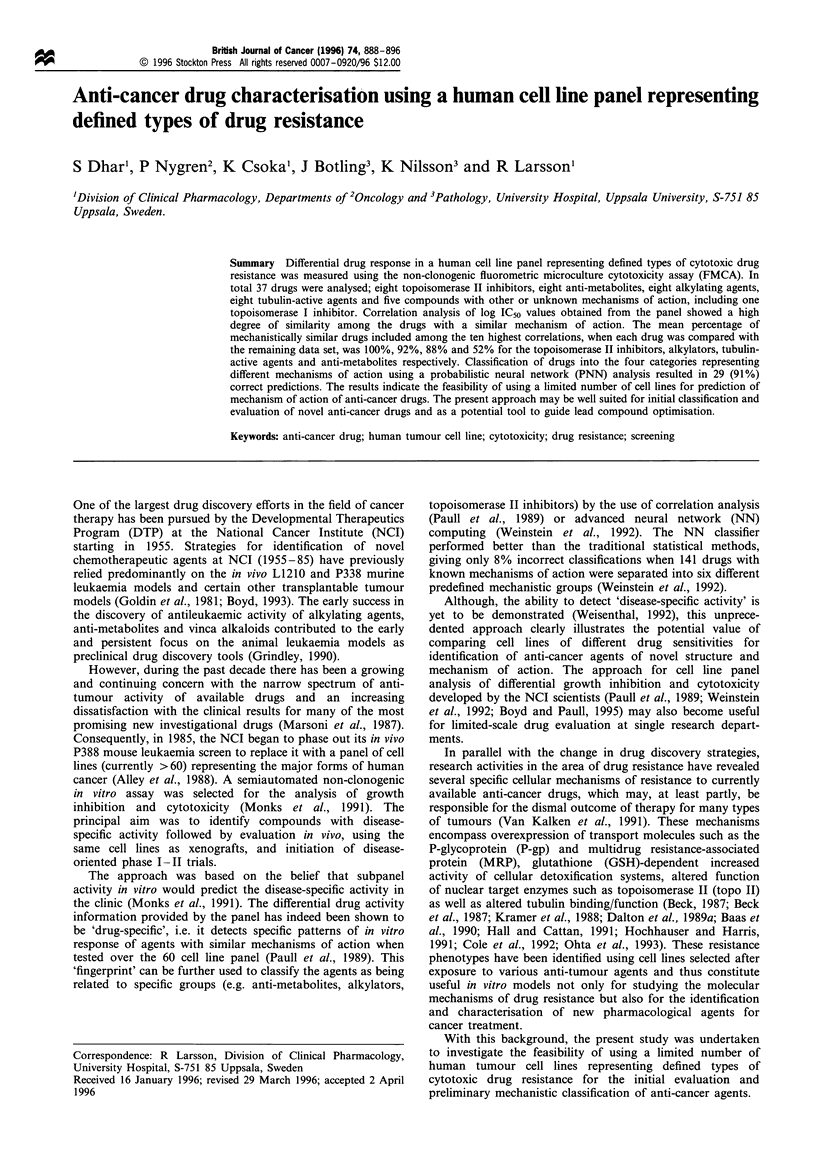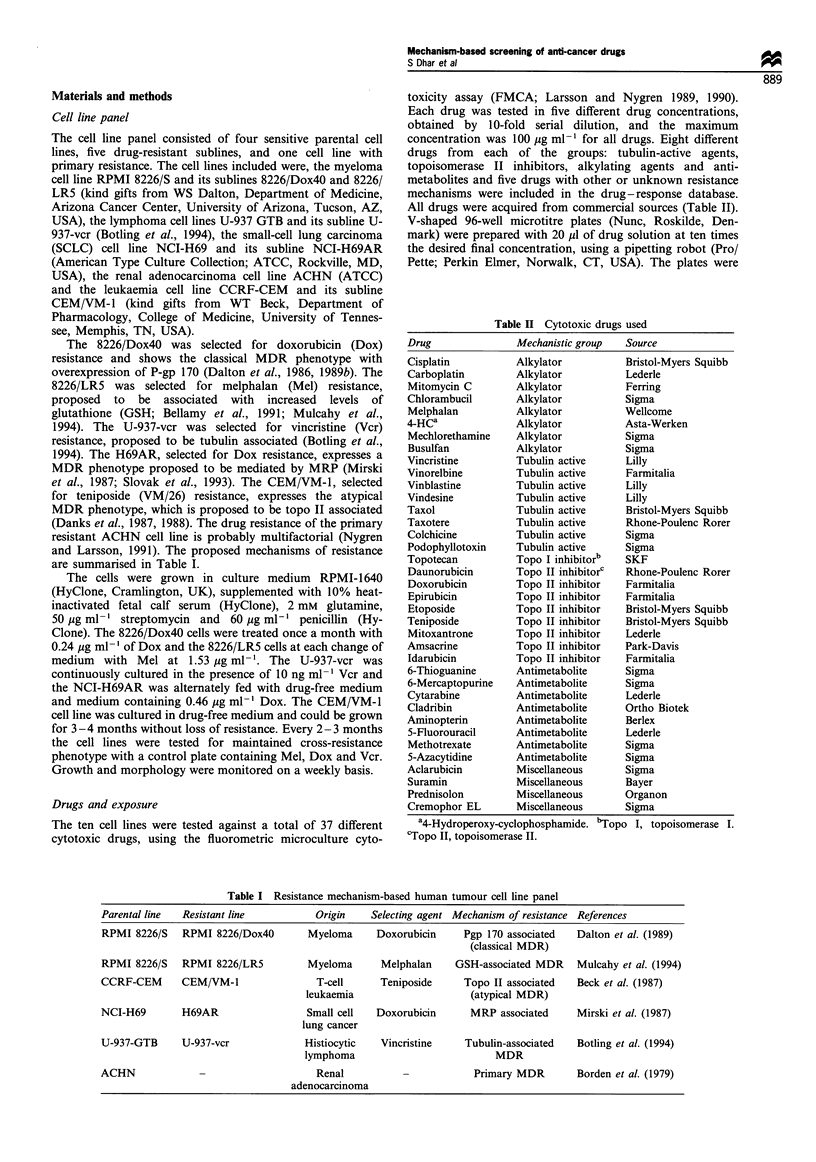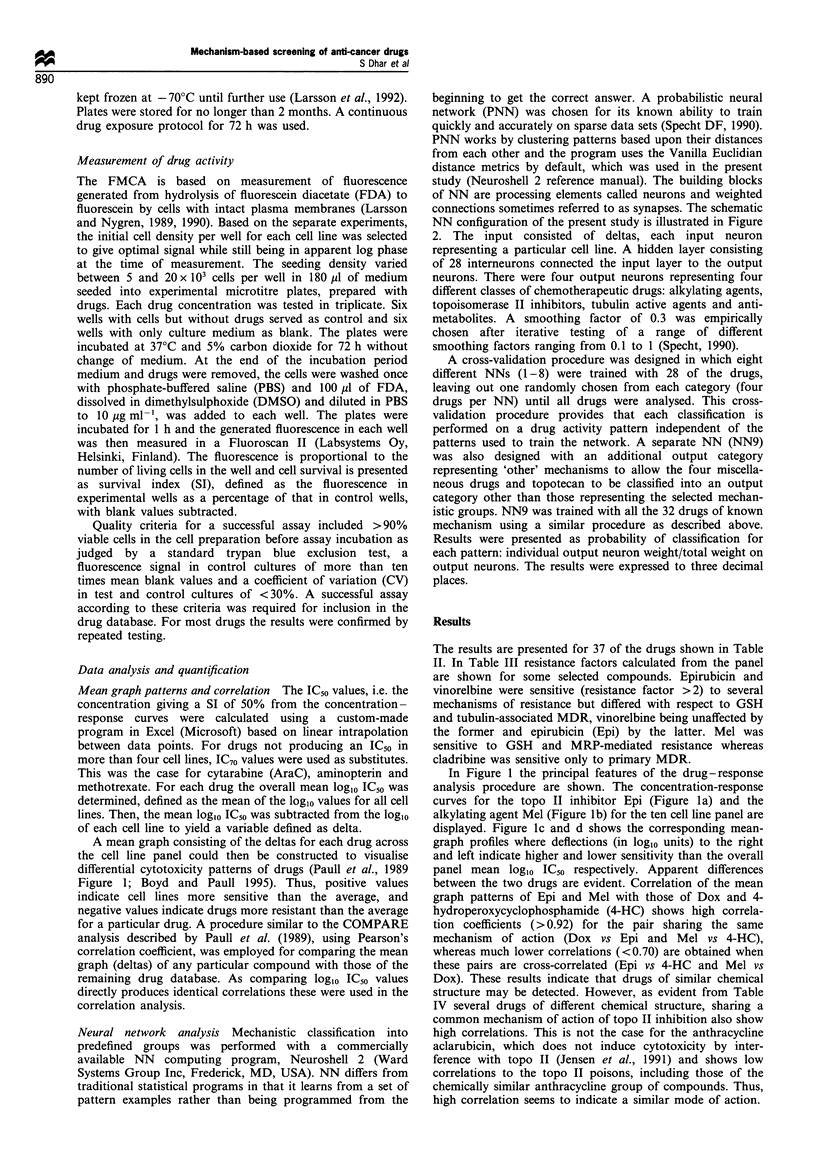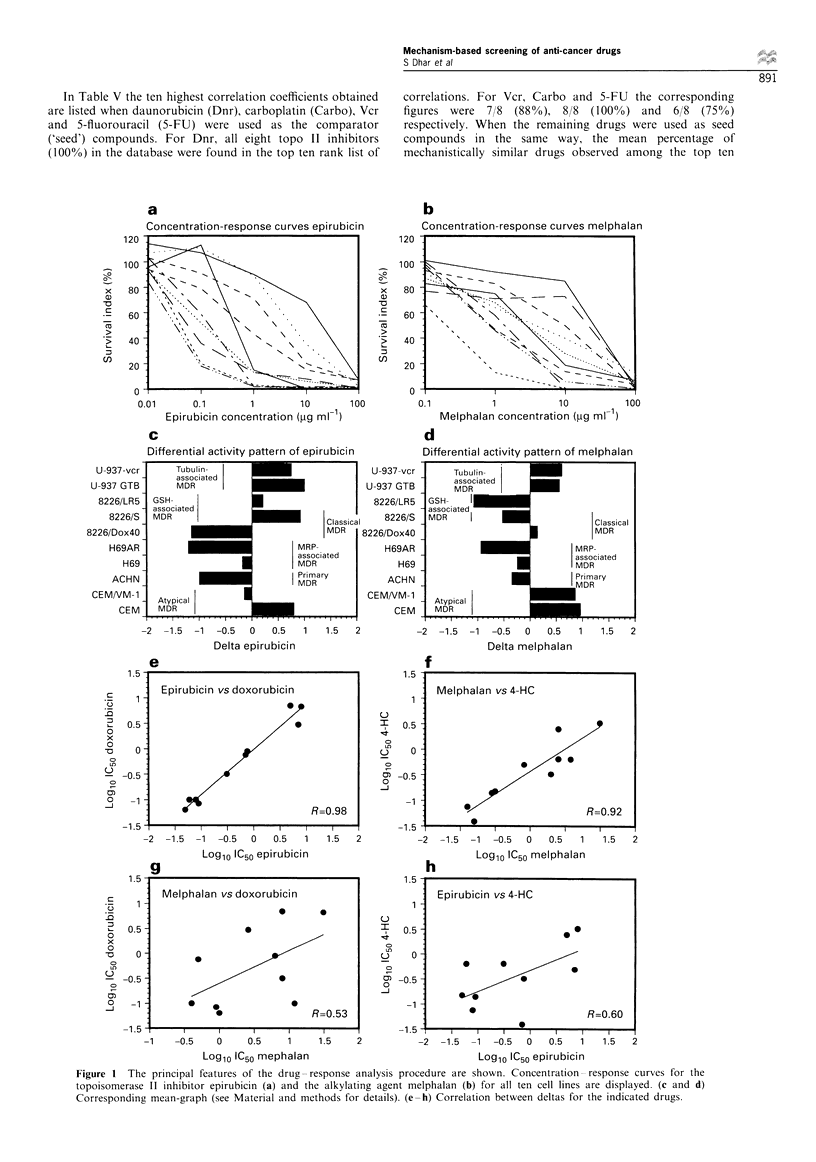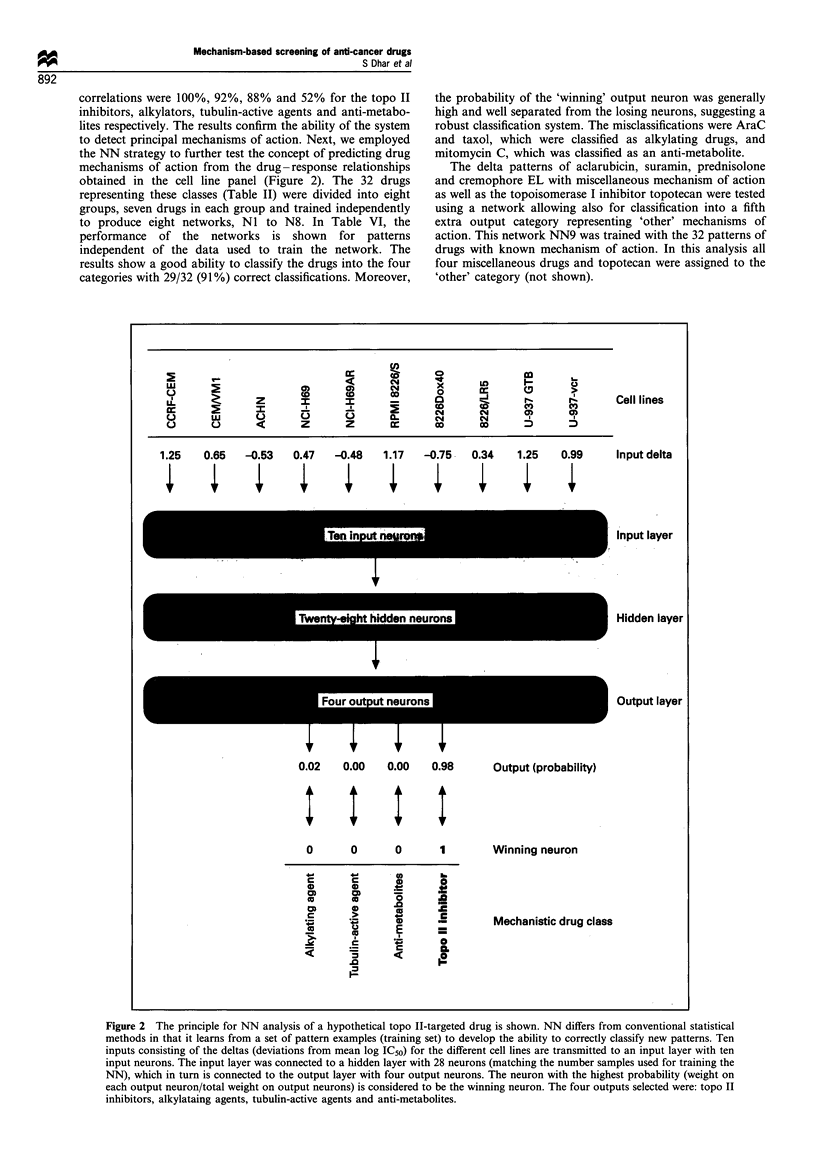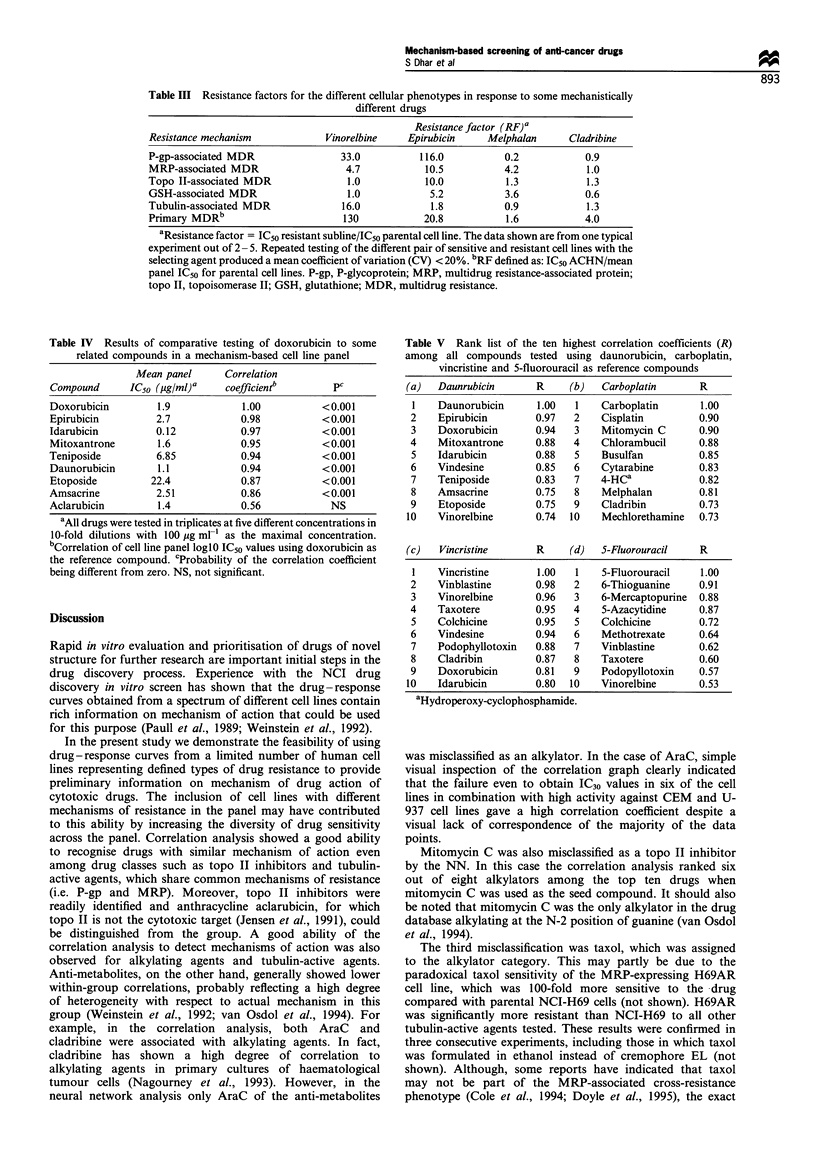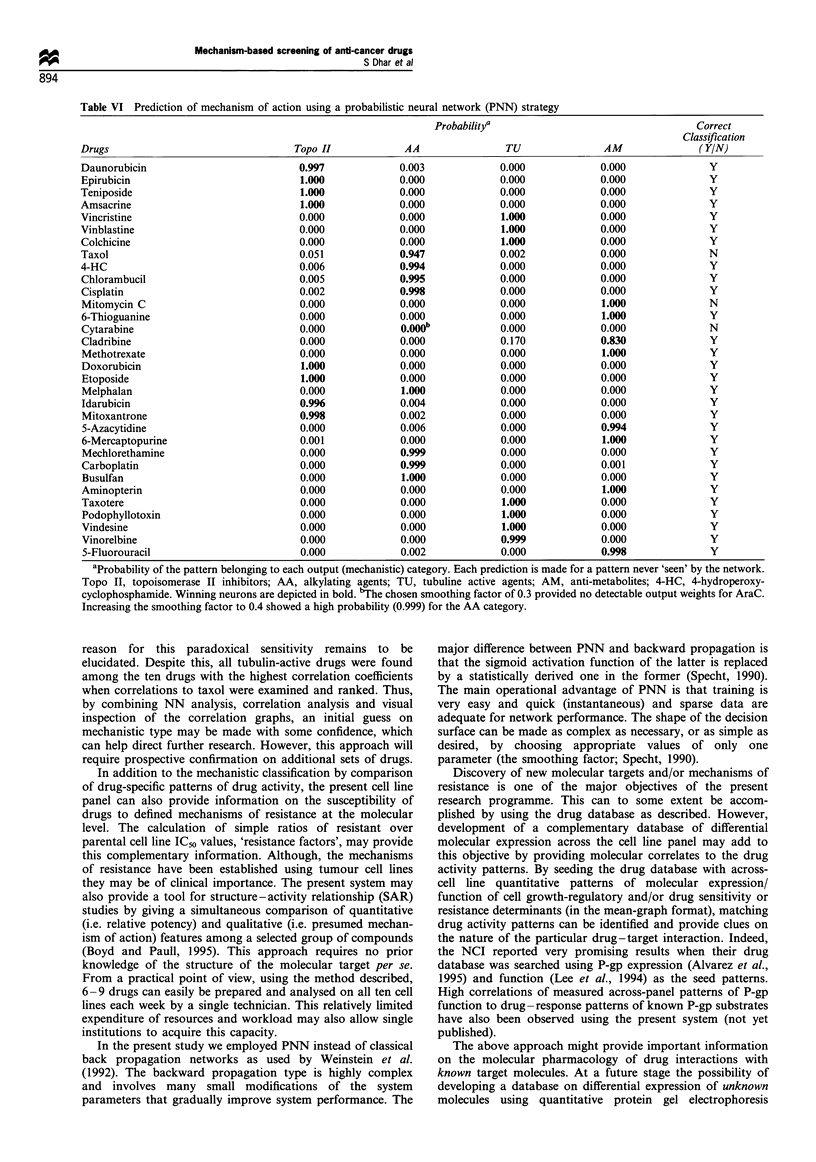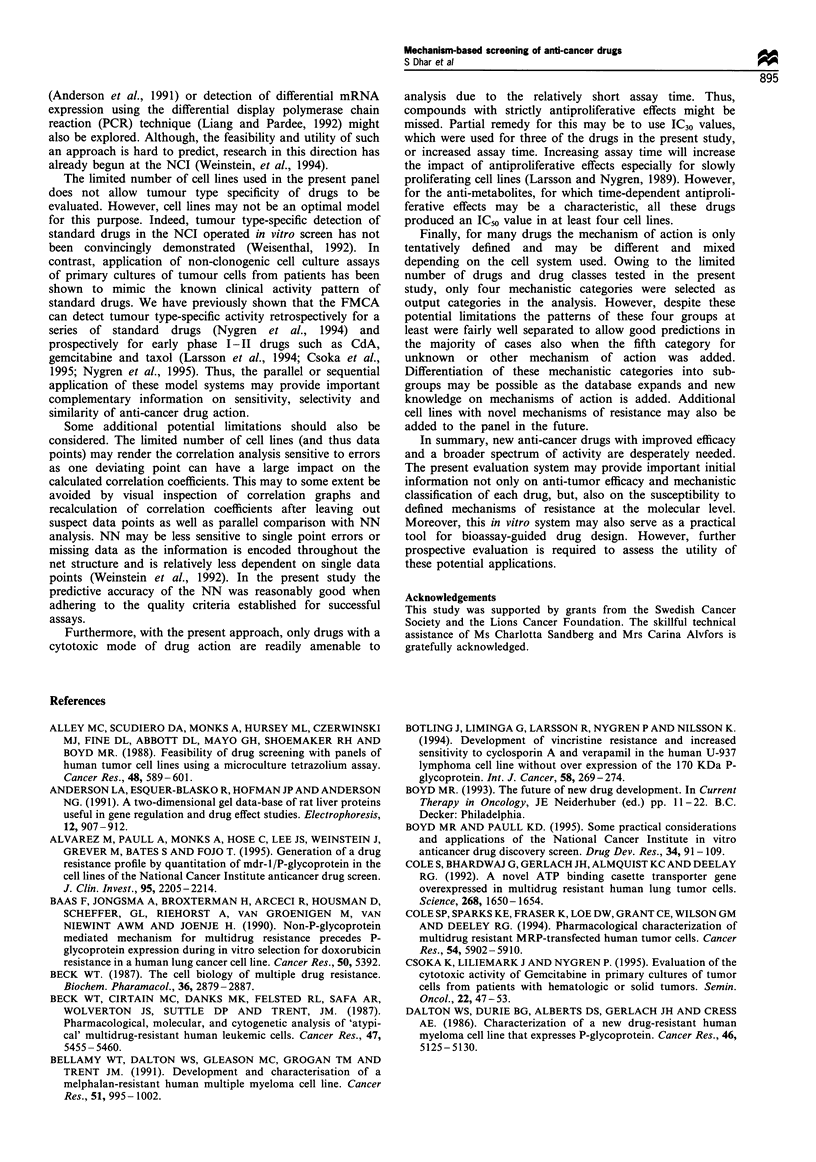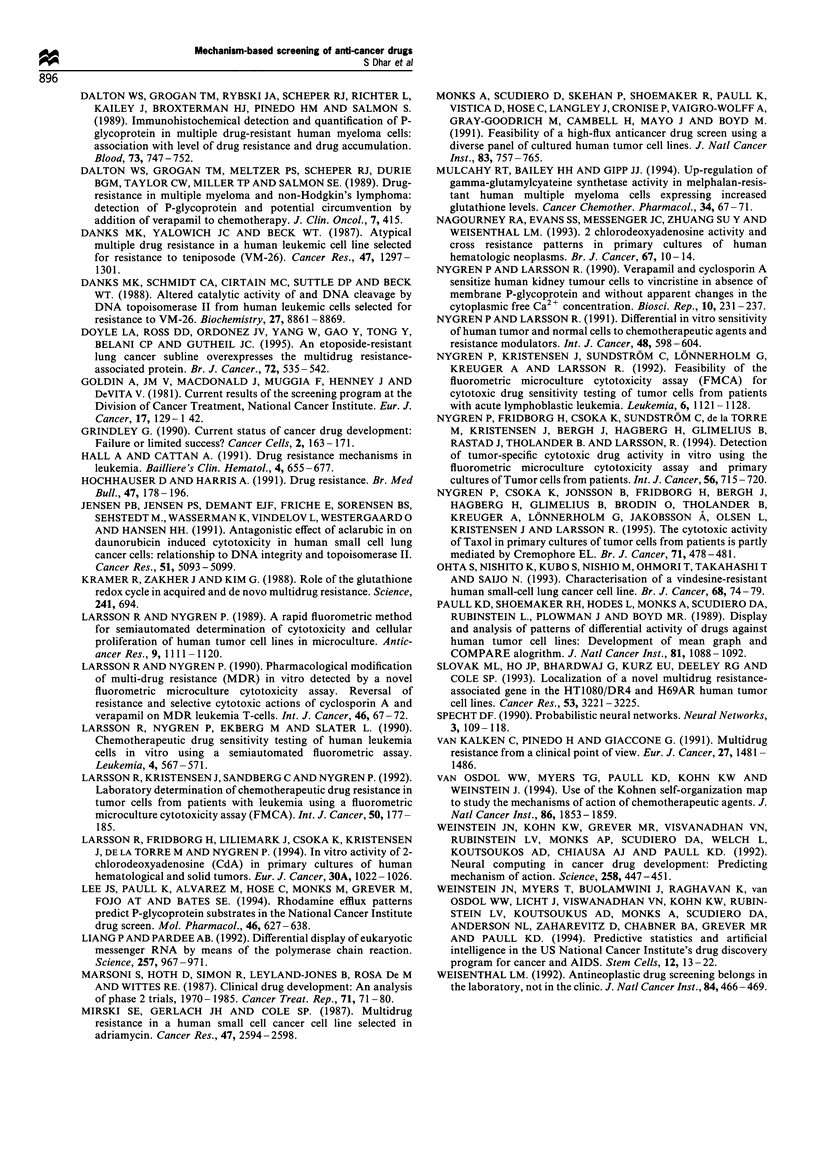# Anti-cancer drug characterisation using a human cell line panel representing defined types of drug resistance.

**DOI:** 10.1038/bjc.1996.453

**Published:** 1996-09

**Authors:** S. Dhar, P. Nygren, K. Csoka, J. Botling, K. Nilsson, R. Larsson

**Affiliations:** Department of Oncology, University Hospital, Uppsala University, Sweden.

## Abstract

**Images:**


British Journal of Cancer (1996) 74, 888-896
© 1996 Stockton Press All rights reserved 0007-0920/96 $12.00

### Anti-cancer drug characterisation using a human cell line panel representing defined types of drug resistance

S Dhar[1], P Nygren[2], K Csoka[1], J Botling[3], K Nilsson[3] and R Larsson[1]

[1]*Division of Clinical Pharmacology, Departments of* [2]*Oncology and* [3]*Pathology, University Hospital, Uppsala University, S-751 85 Uppsala, Sweden.*

**Summary** Differential drug response in a human cell line panel representing defined types of cytotoxic drug resistance was measured using the non-clonogenic fluorometric microculture cytotoxicity assay (FMCA). In total 37 drugs were analysed; eight topoisomerase II inhibitors, eight anti-metabolites, eight alkylating agents, eight tubulin-active agents and five compounds with other or unknown mechanisms of action, including one topoisomerase I inhibitor. Correlation analysis of log $IC_{50}$ values obtained from the panel showed a high degree of similarity among the drugs with a similar mechanism of action. The mean percentage of mechanistically similar drugs included among the ten highest correlations, when each drug was compared with the remaining data set, was 100%, 92%, 88% and 52% for the topoisomerase II inhibitors, alkylators, tubulin-active agents and anti-metabolites respectively. Classification of drugs into the four categories representing different mechanisms of action using a probabilistic neural network (PNN) analysis resulted in 29 (91%) correct predictions. The results indicate the feasibility of using a limited number of cell lines for prediction of mechanism of action of anti-cancer drugs. The present approach may be well suited for initial classification and evaluation of novel anti-cancer drugs and as a potential tool to guide lead compound optimisation.

**Keywords:** anti-cancer drug; human tumour cell line; cytotoxicity; drug resistance; screening

One of the largest drug discovery efforts in the field of cancer therapy has been pursued by the Developmental Therapeutics Program (DTP) at the National Cancer Institute (NCI) starting in 1955. Strategies for identification of novel chemotherapeutic agents at NCI (1955–85) have previously relied predominantly on the *in vivo* L1210 and P338 murine leukaemia models and certain other transplantable tumour models (Goldin *et al.*, 1981; Boyd, 1993). The early success in the discovery of antileukaemic activity of alkylating agents, anti-metabolites and vinca alkaloids contributed to the early and persistent focus on the animal leukaemia models as preclinical drug discovery tools (Grindley, 1990).

However, during the past decade there has been a growing and continuing concern with the narrow spectrum of anti-tumour activity of available drugs and an increasing dissatisfaction with the clinical results for many of the most promising new investigational drugs (Marsoni *et al.*, 1987). Consequently, in 1985, the NCI began to phase out its *in vivo* P388 mouse leukaemia screen to replace it with a panel of cell lines (currently >60) representing the major forms of human cancer (Alley *et al.*, 1988). A semiautomated non-clonogenic *in vitro* assay was selected for the analysis of growth inhibition and cytotoxicity (Monks *et al.*, 1991). The principal aim was to identify compounds with disease-specific activity followed by evaluation *in vivo*, using the same cell lines as xenografts, and initiation of disease-oriented phase I–II trials.

The approach was based on the belief that subpanel activity *in vitro* would predict the disease-specific activity in the clinic (Monks *et al.*, 1991). The differential drug activity information provided by the panel has indeed been shown to be 'drug-specific', i.e. it detects specific patterns of *in vitro* response of agents with similar mechanisms of action when tested over the 60 cell line panel (Paull *et al.*, 1989). This 'fingerprint' can be further used to classify the agents as being related to specific groups (e.g. anti-metabolites, alkylators, topoisomerase II inhibitors) by the use of correlation analysis (Paull *et al.*, 1989) or advanced neural network (NN) computing (Weinstein *et al.*, 1992). The NN classifier performed better than the traditional statistical methods, giving only 8% incorrect classifications when 141 drugs with known mechanisms of action were separated into six different predefined mechanistic groups (Weinstein *et al.*, 1992).

Although, the ability to detect 'disease-specific activity' is yet to be demonstrated (Weisenthal, 1992), this unprecedented approach clearly illustrates the potential value of comparing cell lines of different drug sensitivities for identification of anti-cancer agents of novel structure and mechanism of action. The approach for cell line panel analysis of differential growth inhibition and cytotoxicity developed by the NCI scientists (Paull *et al.*, 1989; Weinstein *et al.*, 1992; Boyd and Paull, 1995) may also become useful for limited-scale drug evaluation at single research departments.

In parallel with the change in drug discovery strategies, research activities in the area of drug resistance have revealed several specific cellular mechanisms of resistance to currently available anti-cancer drugs, which may, at least partly, be responsible for the dismal outcome of therapy for many types of tumours (Van Kalken *et al.*, 1991). These mechanisms encompass overexpression of transport molecules such as the P-glycoprotein (P-gp) and multidrug resistance-associated protein (MRP), glutathione (GSH)-dependent increased activity of cellular detoxification systems, altered function of nuclear target enzymes such as topoisomerase II (topo II) as well as altered tubulin binding/function (Beck, 1987; Beck *et al.*, 1987; Kramer *et al.*, 1988; Dalton *et al.*, 1989*a*; Baas *et al.*, 1990; Hall and Cattan, 1991; Hochhauser and Harris, 1991; Cole *et al.*, 1992; Ohta *et al.*, 1993). These resistance phenotypes have been identified using cell lines selected after exposure to various anti-tumour agents and thus constitute useful *in vitro* models not only for studying the molecular mechanisms of drug resistance but also for the identification and characterisation of new pharmacological agents for cancer treatment.

With this background, the present study was undertaken to investigate the feasibility of using a limited number of human tumour cell lines representing defined types of cytotoxic drug resistance for the initial evaluation and preliminary mechanistic classification of anti-cancer agents.

Correspondence: R Larsson, Division of Clinical Pharmacology, University Hospital, S-751 85 Uppsala, Sweden
Received 16 January 1996; revised 29 March 1996; accepted 2 April 1996

### Materials and methods

### *Cell line panel*

The cell line panel consisted of four sensitive parental cell lines, five drug-resistant sublines, and one cell line with primary resistance. The cell lines included were, the myeloma cell line RPMI 8226/S and its sublines 8226/Dox40 and 8226/LR5 (kind gifts from WS Dalton, Department of Medicine, Arizona Cancer Center, University of Arizona, Tucson, AZ, USA), the lymphoma cell lines U-937 GTB and its subline U-937-vcr (Botling *et al.*, 1994), the small-cell lung carcinoma (SCLC) cell line NCI-H69 and its subline NCI-H69AR (American Type Culture Collection; ATCC, Rockville, MD, USA), the renal adenocarcinoma cell line ACHN (ATCC) and the leukaemia cell line CCRF-CEM and its subline CEM/VM-1 (kind gifts from WT Beck, Department of Pharmacology, College of Medicine, University of Tennessee, Memphis, TN, USA).

The 8226/Dox40 was selected for doxorubicin (Dox) resistance and shows the classical MDR phenotype with overexpression of P-gp 170 (Dalton *et al.*, 1986, 1989*b*). The 8226/LR5 was selected for melphalan (Mel) resistance, proposed to be associated with increased levels of glutathione (GSH; Bellamy *et al.*, 1991; Mulcahy *et al.*, 1994). The U-937-vcr was selected for vincristine (Vcr) resistance, proposed to be tubulin associated (Botling *et al.*, 1994). The H69AR, selected for Dox resistance, expresses a MDR phenotype proposed to be mediated by MRP (Mirski *et al.*, 1987; Slovak *et al.*, 1993). The CEM/VM-1, selected for teniposide (VM/26) resistance, expresses the atypical MDR phenotype, which is proposed to be topo II associated (Danks *et al.*, 1987, 1988). The drug resistance of the primary resistant ACHN cell line is probably multifactorial (Nygren and Larsson, 1991). The proposed mechanisms of resistance are summarised in Table I.

The cells were grown in culture medium RPMI-1640 (HyClone, Cramlington, UK), supplemented with 10% heat-inactivated fetal calf serum (HyClone), 2 mM glutamine, 50 $\mu g\ ml^{-1}$ streptomycin and 60 $\mu g\ ml^{-1}$ penicillin (HyClone). The 8226/Dox40 cells were treated once a month with 0.24 $\mu g\ ml^{-1}$ of Dox and the 8226/LR5 cells at each change of medium with Mel at 1.53 $\mu g\ ml^{-1}$. The U-937-vcr was continuously cultured in the presence of 10 ng $ml^{-1}$ Vcr and the NCI-H69AR was alternately fed with drug-free medium and medium containing 0.46 $\mu g\ ml^{-1}$ Dox. The CEM/VM-1 cell line was cultured in drug-free medium and could be grown for 3–4 months without loss of resistance. Every 2–3 months the cell lines were tested for maintained cross-resistance phenotype with a control plate containing Mel, Dox and Vcr. Growth and morphology were monitored on a weekly basis.

### *Drugs and exposure*

The ten cell lines were tested against a total of 37 different cytotoxic drugs, using the fluorometric microculture cytotoxicity assay (FMCA; Larsson and Nygren 1989, 1990). Each drug was tested in five different drug concentrations, obtained by 10-fold serial dilution, and the maximum concentration was 100 $\mu g\ ml^{-1}$ for all drugs. Eight different drugs from each of the groups: tubulin-active agents, topoisomerase II inhibitors, alkylating agents and antimetabolites and five drugs with other or unknown resistance mechanisms were included in the drug–response database. All drugs were acquired from commercial sources (Table II). V-shaped 96-well microtitre plates (Nunc, Roskilde, Denmark) were prepared with 20 $\mu l$ of drug solution at ten times the desired final concentration, using a pipetting robot (Pro/Pette; Perkin Elmer, Norwalk, CT, USA). The plates were

**Table II** Cytotoxic drugs used

| *Drug* | *Mechanistic group* | *Source* |
|---|---|---|
| Cisplatin | Alkylator | Bristol-Myers Squibb |
| Carboplatin | Alkylator | Lederle |
| Mitomycin C | Alkylator | Ferring |
| Chlorambucil | Alkylator | Sigma |
| Melphalan | Alkylator | Wellcome |
| 4-HC[a] | Alkylator | Asta-Werken |
| Mechlorethamine | Alkylator | Sigma |
| Busulfan | Alkylator | Sigma |
| Vincristine | Tubulin active | Lilly |
| Vinorelbine | Tubulin active | Farmitalia |
| Vinblastine | Tubulin active | Lilly |
| Vindesine | Tubulin active | Lilly |
| Taxol | Tubulin active | Bristol-Myers Squibb |
| Taxotere | Tubulin active | Rhone-Poulenc Rorer |
| Colchicine | Tubulin active | Sigma |
| Podophyllotoxin | Tubulin active | Sigma |
| Topotecan | Topo I inhibitor[b] | SKF |
| Daunorubicin | Topo II inhibitor[c] | Rhone-Poulenc Rorer |
| Doxorubicin | Topo II inhibitor | Farmitalia |
| Epirubicin | Topo II inhibitor | Farmitalia |
| Etoposide | Topo II inhibitor | Bristol-Myers Squibb |
| Teniposide | Topo II inhibitor | Bristol-Myers Squibb |
| Mitoxantrone | Topo II inhibitor | Lederle |
| Amsacrine | Topo II inhibitor | Park-Davis |
| Idarubicin | Topo II inhibitor | Farmitalia |
| 6-Thioguanine | Antimetabolite | Sigma |
| 6-Mercaptopurine | Antimetabolite | Sigma |
| Cytarabine | Antimetabolite | Lederle |
| Cladribin | Antimetabolite | Ortho Biotek |
| Aminopterin | Antimetabolite | Berlex |
| 5-Fluorouracil | Antimetabolite | Lederle |
| Methotrexate | Antimetabolite | Sigma |
| 5-Azacytidine | Antimetabolite | Sigma |
| Aclarubicin | Miscellaneous | Sigma |
| Suramin | Miscellaneous | Bayer |
| Prednisolon | Miscellaneous | Organon |
| Cremophor EL | Miscellaneous | Sigma |

[a]4-Hydroperoxy-cyclophosphamide. [b]Topo I, topoisomerase I. [c]Topo II, topoisomerase II.

**Table I** Resistance mechanism-based human tumour cell line panel

| *Parental line* | *Resistant line* | *Origin* | *Selecting agent* | *Mechanism of resistance* | *References* |
|---|---|---|---|---|---|
| RPMI 8226/S | RPMI 8226/Dox40 | Myeloma | Doxorubicin | Pgp 170 associated (classical MDR) | Dalton *et al.* (1989) |
| RPMI 8226/S | RPMI 8226/LR5 | Myeloma | Melphalan | GSH-associated MDR | Mulcahy *et al.* (1994) |
| CCRF-CEM | CEM/VM-1 | T-cell leukaemia | Teniposide | Topo II associated (atypical MDR) | Beck *et al.* (1987) |
| NCI-H69 | H69AR | Small cell lung cancer | Doxorubicin | MRP associated | Mirski *et al.* (1987) |
| U-937-GTB | U-937-vcr | Histiocytic lymphoma | Vincristine | Tubulin-associated MDR | Botling *et al.* (1994) |
| ACHN | – | Renal adenocarcinoma | – | Primary MDR | Borden *et al.* (1979) |

kept frozen at −70°C until further use (Larsson et al., 1992). Plates were stored for no longer than 2 months. A continuous drug exposure protocol for 72 h was used.

*Measurement of drug activity*

The FMCA is based on measurement of fluorescence generated from hydrolysis of fluorescein diacetate (FDA) to fluorescein by cells with intact plasma membranes (Larsson and Nygren, 1989, 1990). Based on the separate experiments, the initial cell density per well for each cell line was selected to give optimal signal while still being in apparent log phase at the time of measurement. The seeding density varied between 5 and $20 \times 10^3$ cells per well in 180 $\mu l$ of medium seeded into experimental microtitre plates, prepared with drugs. Each drug concentration was tested in triplicate. Six wells with cells but without drugs served as control and six wells with only culture medium as blank. The plates were incubated at 37°C and 5% carbon dioxide for 72 h without change of medium. At the end of the incubation period medium and drugs were removed, the cells were washed once with phosphate-buffered saline (PBS) and 100 $\mu l$ of FDA, dissolved in dimethylsulphoxide (DMSO) and diluted in PBS to 10 $\mu g\ ml^{-1}$, was added to each well. The plates were incubated for 1 h and the generated fluorescence in each well was then measured in a Fluoroscan II (Labsystems Oy, Helsinki, Finland). The fluorescence is proportional to the number of living cells in the well and cell survival is presented as survival index (SI), defined as the fluorescence in experimental wells as a percentage of that in control wells, with blank values subtracted.

Quality criteria for a successful assay included >90% viable cells in the cell preparation before assay incubation as judged by a standard trypan blue exclusion test, a fluorescence signal in control cultures of more than ten times mean blank values and a coefficient of variation (CV) in test and control cultures of <30%. A successful assay according to these criteria was required for inclusion in the drug database. For most drugs the results were confirmed by repeated testing.

*Data analysis and quantification*

*Mean graph patterns and correlation* The $IC_{50}$ values, i.e. the concentration giving a SI of 50% from the concentration–response curves were calculated using a custom-made program in Excel (Microsoft) based on linear intrapolation between data points. For drugs not producing an $IC_{50}$ in more than four cell lines, $IC_{70}$ values were used as substitutes. This was the case for cytarabine (AraC), aminopterin and methotrexate. For each drug the overall mean $\log_{10}$ $IC_{50}$ was determined, defined as the mean of the $\log_{10}$ values for all cell lines. Then, the mean $\log_{10}$ $IC_{50}$ was subtracted from the $\log_{10}$ of each cell line to yield a variable defined as delta.

A mean graph consisting of the deltas for each drug across the cell line panel could then be constructed to visualise differential cytotoxicity patterns of drugs (Paull et al., 1989 Figure 1; Boyd and Paull 1995). Thus, positive values indicate cell lines more sensitive than the average, and negative values indicate drugs more resistant than the average for a particular drug. A procedure similar to the COMPARE analysis described by Paull et al. (1989), using Pearson's correlation coefficient, was employed for comparing the mean graph (deltas) of any particular compound with those of the remaining drug database. As comparing $\log_{10}$ $IC_{50}$ values directly produces identical correlations these were used in the correlation analysis.

*Neural network analysis* Mechanistic classification into predefined groups was performed with a commercially available NN computing program, Neuroshell 2 (Ward Systems Group Inc, Frederick, MD, USA). NN differs from traditional statistical programs in that it learns from a set of pattern examples rather than being programmed from the beginning to get the correct answer. A probabilistic neural network (PNN) was chosen for its known ability to train quickly and accurately on sparse data sets (Specht DF, 1990). PNN works by clustering patterns based upon their distances from each other and the program uses the Vanilla Euclidian distance metrics by default, which was used in the present study (Neuroshell 2 reference manual). The building blocks of NN are processing elements called neurons and weighted connections sometimes referred to as synapses. The schematic NN configuration of the present study is illustrated in Figure 2. The input consisted of deltas, each input neuron representing a particular cell line. A hidden layer consisting of 28 interneurons connected the input layer to the output neurons. There were four output neurons representing four different classes of chemotherapeutic drugs: alkylating agents, topoisomerase II inhibitors, tubulin active agents and anti-metabolites. A smoothing factor of 0.3 was empirically chosen after iterative testing of a range of different smoothing factors ranging from 0.1 to 1 (Specht, 1990).

A cross-validation procedure was designed in which eight different NNs (1–8) were trained with 28 of the drugs, leaving out one randomly chosen from each category (four drugs per NN) until all drugs were analysed. This cross-validation procedure provides that each classification is performed on a drug activity pattern independent of the patterns used to train the network. A separate NN (NN9) was also designed with an additional output category representing 'other' mechanisms to allow the four miscellaneous drugs and topotecan to be classified into an output category other than those representing the selected mechanistic groups. NN9 was trained with all the 32 drugs of known mechanism using a similar procedure as described above. Results were presented as probability of classification for each pattern: individual output neuron weight/total weight on output neurons. The results were expressed to three decimal places.

### Results

The results are presented for 37 of the drugs shown in Table II. In Table III resistance factors calculated from the panel are shown for some selected compounds. Epirubicin and vinorelbine were sensitive (resistance factor >2) to several mechanisms of resistance but differed with respect to GSH and tubulin-associated MDR, vinorelbine being unaffected by the former and epirubicin (Epi) by the latter. Mel was sensitive to GSH and MRP-mediated resistance whereas cladribine was sensitive only to primary MDR.

In Figure 1 the principal features of the drug–response analysis procedure are shown. The concentration-response curves for the topo II inhibitor Epi (Figure 1a) and the alkylating agent Mel (Figure 1b) for the ten cell line panel are displayed. Figure 1c and d shows the corresponding mean-graph profiles where deflections (in $\log_{10}$ units) to the right and left indicate higher and lower sensitivity than the overall panel mean $\log_{10}$ $IC_{50}$ respectively. Apparent differences between the two drugs are evident. Correlation of the mean graph patterns of Epi and Mel with those of Dox and 4-hydroperoxycyclophosphamide (4-HC) shows high correlation coefficients (>0.92) for the pair sharing the same mechanism of action (Dox *vs* Epi and Mel *vs* 4-HC), whereas much lower correlations (<0.70) are obtained when these pairs are cross-correlated (Epi *vs* 4-HC and Mel *vs* Dox). These results indicate that drugs of similar chemical structure may be detected. However, as evident from Table IV several drugs of different chemical structure, sharing a common mechanism of action of topo II inhibition also show high correlations. This is not the case for the anthracycline aclarubicin, which does not induce cytotoxicity by interference with topo II (Jensen et al., 1991) and shows low correlations to the topo II poisons, including those of the chemically similar anthracycline group of compounds. Thus, high correlation seems to indicate a similar mode of action.

In Table V the ten highest correlation coefficients obtained are listed when daunorubicin (Dnr), carboplatin (Carbo), Vcr and 5-fluorouracil (5-FU) were used as the comparator ('seed') compounds. For Dnr, all eight topo II inhibitors (100%) in the database were found in the top ten rank list of correlations. For Vcr, Carbo and 5-FU the corresponding figures were 7/8 (88%), 8/8 (100%) and 6/8 (75%) respectively. When the remaining drugs were used as seed compounds in the same way, the mean percentage of mechanistically similar drugs observed among the top ten

**Figure 1** The principal features of the drug–response analysis procedure are shown. Concentration–response curves for the topoisomerase II inhibitor epirubicin (**a**) and the alkylating agent melphalan (**b**) for all ten cell lines are displayed. (**c** and **d**) Corresponding mean-graph (see Material and methods for details). (**e**–**h**) Correlation between deltas for the indicated drugs.

correlations were 100%, 92%, 88% and 52% for the topo II inhibitors, alkylators, tubulin-active agents and anti-metabolites respectively. The results confirm the ability of the system to detect principal mechanisms of action. Next, we employed the NN strategy to further test the concept of predicting drug mechanisms of action from the drug–response relationships obtained in the cell line panel (Figure 2). The 32 drugs representing these classes (Table II) were divided into eight groups, seven drugs in each group and trained independently to produce eight networks, N1 to N8. In Table VI, the performance of the networks is shown for patterns independent of the data used to train the network. The results show a good ability to classify the drugs into the four categories with 29/32 (91%) correct classifications. Moreover, the probability of the 'winning' output neuron was generally high and well separated from the losing neurons, suggesting a robust classification system. The misclassifications were AraC and taxol, which were classified as alkylating drugs, and mitomycin C, which was classified as an anti-metabolite.

The delta patterns of aclarubicin, suramin, prednisolone and cremophore EL with miscellaneous mechanism of action as well as the topoisomerase I inhibitor topotecan were tested using a network allowing also for classification into a fifth extra output category representing 'other' mechanisms of action. This network NN9 was trained with the 32 patterns of drugs with known mechanism of action. In this analysis all four miscellaneous drugs and topotecan were assigned to the 'other' category (not shown).

**Figure 2** The principle for NN analysis of a hypothetical topo II-targeted drug is shown. NN differs from conventional statistical methods in that it learns from a set of pattern examples (training set) to develop the ability to correctly classify new patterns. Ten inputs consisting of the deltas (deviations from mean log $IC_{50}$) for the different cell lines are transmitted to an input layer with ten input neurons. The input layer was connected to a hidden layer with 28 neurons (matching the number samples used for training the NN), which in turn is connected to the output layer with four output neurons. The neuron with the highest probability (weight on each output neuron/total weight on output neurons) is considered to be the winning neuron. The four outputs selected were: topo II inhibitors, alkylataing agents, tubulin-active agents and anti-metabolites.

Table III Resistance factors for the different cellular phenotypes in response to some mechanistically different drugs

| Resistance mechanism | Vinorelbine | Resistance factor (RF)[a] Epirubicin | Melphalan | Cladribine |
|---|---|---|---|---|
| P-gp-associated MDR | 33.0 | 116.0 | 0.2 | 0.9 |
| MRP-associated MDR | 4.7 | 10.5 | 4.2 | 1.0 |
| Topo II-associated MDR | 1.0 | 10.0 | 1.3 | 1.3 |
| GSH-associated MDR | 1.0 | 5.2 | 3.6 | 0.6 |
| Tubulin-associated MDR | 16.0 | 1.8 | 0.9 | 1.3 |
| Primary MDR[b] | 130 | 20.8 | 1.6 | 4.0 |

[a]Resistance factor = $IC_{50}$ resistant subline/$IC_{50}$ parental cell line. The data shown are from one typical experiment out of 2–5. Repeated testing of the different pair of sensitive and resistant cell lines with the selecting agent produced a mean coefficient of variation (CV) <20%. [b]RF defined as: $IC_{50}$ ACHN/mean panel $IC_{50}$ for parental cell lines. P-gp, P-glycoprotein; MRP, multidrug resistance-associated protein; topo II, topoisomerase II; GSH, glutathione; MDR, multidrug resistance.

Table IV Results of comparative testing of doxorubicin to some related compounds in a mechanism-based cell line panel

| Compound | Mean panel $IC_{50}$ ($\mu$g/ml)[a] | Correlation coefficient[b] | P[c] |
|---|---|---|---|
| Doxorubicin | 1.9 | 1.00 | <0.001 |
| Epirubicin | 2.7 | 0.98 | <0.001 |
| Idarubicin | 0.12 | 0.97 | <0.001 |
| Mitoxantrone | 1.6 | 0.95 | <0.001 |
| Teniposide | 6.85 | 0.94 | <0.001 |
| Daunorubicin | 1.1 | 0.94 | <0.001 |
| Etoposide | 22.4 | 0.87 | <0.001 |
| Amsacrine | 2.51 | 0.86 | <0.001 |
| Aclarubicin | 1.4 | 0.56 | NS |

[a]All drugs were tested in triplicates at five different concentrations in 10-fold dilutions with 100 $\mu$g $ml^{-1}$ as the maximal concentration. [b]Correlation of cell line panel log10 $IC_{50}$ values using doxorubicin as the reference compound. [c]Probability of the correlation coefficient being different from zero. NS, not significant.

Table V Rank list of the ten highest correlation coefficients (*R*) among all compounds tested using daunorubicin, carboplatin, vincristine and 5-fluorouracil as reference compounds

| (a) | Daunrubicin | R | (b) | Carboplatin | R |
|---|---|---|---|---|---|
| 1 | Daunorubicin | 1.00 | 1 | Carboplatin | 1.00 |
| 2 | Epirubicin | 0.97 | 2 | Cisplatin | 0.90 |
| 3 | Doxorubicin | 0.94 | 3 | Mitomycin C | 0.90 |
| 4 | Mitoxantrone | 0.88 | 4 | Chlorambucil | 0.88 |
| 5 | Idarubicin | 0.88 | 5 | Busulfan | 0.85 |
| 6 | Vindesine | 0.85 | 6 | Cytarabine | 0.83 |
| 7 | Teniposide | 0.83 | 7 | 4-HC[a] | 0.82 |
| 8 | Amsacrine | 0.75 | 8 | Melphalan | 0.81 |
| 9 | Etoposide | 0.75 | 9 | Cladribin | 0.73 |
| 10 | Vinorelbine | 0.74 | 10 | Mechlorethamine | 0.73 |

| (c) | Vincristine | R | (d) | 5-Fluorouracil | R |
|---|---|---|---|---|---|
| 1 | Vincristine | 1.00 | 1 | 5-Fluorouracil | 1.00 |
| 2 | Vinblastine | 0.98 | 2 | 6-Thioguanine | 0.91 |
| 3 | Vinorelbine | 0.96 | 3 | 6-Mercaptopurine | 0.88 |
| 4 | Taxotere | 0.95 | 4 | 5-Azacytidine | 0.87 |
| 5 | Colchicine | 0.95 | 5 | Colchicine | 0.72 |
| 6 | Vindesine | 0.94 | 6 | Methotrexate | 0.64 |
| 7 | Podophyllotoxin | 0.88 | 7 | Vinblastine | 0.62 |
| 8 | Cladribin | 0.87 | 8 | Taxotere | 0.60 |
| 9 | Doxorubicin | 0.81 | 9 | Podopyllotoxin | 0.57 |
| 10 | Idarubicin | 0.80 | 10 | Vinorelbine | 0.53 |

[a]Hydroperoxy-cyclophosphamide.

### Discussion

Rapid *in vitro* evaluation and prioritisation of drugs of novel structure for further research are important initial steps in the drug discovery process. Experience with the NCI drug discovery *in vitro* screen has shown that the drug–response curves obtained from a spectrum of different cell lines contain rich information on mechanism of action that could be used for this purpose (Paull *et al.*, 1989; Weinstein *et al.*, 1992).

In the present study we demonstrate the feasibility of using drug–response curves from a limited number of human cell lines representing defined types of drug resistance to provide preliminary information on mechanism of drug action of cytotoxic drugs. The inclusion of cell lines with different mechanisms of resistance in the panel may have contributed to this ability by increasing the diversity of drug sensitivity across the panel. Correlation analysis showed a good ability to recognise drugs with similar mechanism of action even among drug classes such as topo II inhibitors and tubulin-active agents, which share common mechanisms of resistance (i.e. P-gp and MRP). Moreover, topo II inhibitors were readily identified and anthracycline aclarubicin, for which topo II is not the cytotoxic target (Jensen *et al.*, 1991), could be distinguished from the group. A good ability of the correlation analysis to detect mechanisms of action was also observed for alkylating agents and tubulin-active agents. Anti-metabolites, on the other hand, generally showed lower within-group correlations, probably reflecting a high degree of heterogeneity with respect to actual mechanism in this group (Weinstein *et al.*, 1992; van Osdol *et al.*, 1994). For example, in the correlation analysis, both AraC and cladribine were associated with alkylating agents. In fact, cladribine has shown a high degree of correlation to alkylating agents in primary cultures of haematological tumour cells (Nagourney *et al.*, 1993). However, in the neural network analysis only AraC of the anti-metabolites was misclassified as an alkylator. In the case of AraC, simple visual inspection of the correlation graph clearly indicated that the failure even to obtain $IC_{30}$ values in six of the cell lines in combination with high activity against CEM and U-937 cell lines gave a high correlation coefficient despite a visual lack of correspondence of the majority of the data points.

Mitomycin C was also misclassified as a topo II inhibitor by the NN. In this case the correlation analysis ranked six out of eight alkylators among the top ten drugs when mitomycin C was used as the seed compound. It should also be noted that mitomycin C was the only alkylator in the drug database alkylating at the N-2 position of guanine (van Osdol *et al.*, 1994).

The third misclassification was taxol, which was assigned to the alkylator category. This may partly be due to the paradoxical taxol sensitivity of the MRP-expressing H69AR cell line, which was 100-fold more sensitive to the drug compared with parental NCI-H69 cells (not shown). H69AR was significantly more resistant than NCI-H69 to all other tubulin-active agents tested. These results were confirmed in three consecutive experiments, including those in which taxol was formulated in ethanol instead of cremophore EL (not shown). Although, some reports have indicated that taxol may not be part of the MRP-associated cross-resistance phenotype (Cole *et al.*, 1994; Doyle *et al.*, 1995), the exact

**Table VI** Prediction of mechanism of action using a probabilistic neural network (PNN) strategy

| Drugs | Probability[a] Topo II | AA | TU | AM | Correct Classification (Y/N) |
|---|---|---|---|---|---|
| Daunorubicin | **0.997** | 0.003 | 0.000 | 0.000 | Y |
| Epirubicin | **1.000** | 0.000 | 0.000 | 0.000 | Y |
| Teniposide | **1.000** | 0.000 | 0.000 | 0.000 | Y |
| Amsacrine | **1.000** | 0.000 | 0.000 | 0.000 | Y |
| Vincristine | 0.000 | 0.000 | **1.000** | 0.000 | Y |
| Vinblastine | 0.000 | 0.000 | **1.000** | 0.000 | Y |
| Colchicine | 0.000 | 0.000 | **1.000** | 0.000 | Y |
| Taxol | 0.051 | **0.947** | 0.002 | 0.000 | N |
| 4-HC | 0.006 | **0.994** | 0.000 | 0.000 | Y |
| Chlorambucil | 0.005 | **0.995** | 0.000 | 0.000 | Y |
| Cisplatin | 0.002 | **0.998** | 0.000 | 0.000 | Y |
| Mitomycin C | 0.000 | 0.000 | 0.000 | **1.000** | N |
| 6-Thioguanine | 0.000 | 0.000 | 0.000 | **1.000** | Y |
| Cytarabine | 0.000 | **0.000**[b] | 0.000 | 0.000 | N |
| Cladribine | 0.000 | 0.000 | 0.170 | **0.830** | Y |
| Methotrexate | 0.000 | 0.000 | 0.000 | **1.000** | Y |
| Doxorubicin | **1.000** | 0.000 | 0.000 | 0.000 | Y |
| Etoposide | **1.000** | 0.000 | 0.000 | 0.000 | Y |
| Melphalan | 0.000 | **1.000** | 0.000 | 0.000 | Y |
| Idarubicin | **0.996** | 0.004 | 0.000 | 0.000 | Y |
| Mitoxantrone | **0.998** | 0.002 | 0.000 | 0.000 | Y |
| 5-Azacytidine | 0.000 | 0.006 | 0.000 | **0.994** | Y |
| 6-Mercaptopurine | 0.001 | 0.000 | 0.000 | **1.000** | Y |
| Mechlorethamine | 0.000 | **0.999** | 0.000 | 0.000 | Y |
| Carboplatin | 0.000 | **0.999** | 0.000 | 0.001 | Y |
| Busulfan | 0.000 | **1.000** | 0.000 | 0.000 | Y |
| Aminopterin | 0.000 | 0.000 | 0.000 | **1.000** | Y |
| Taxotere | 0.000 | 0.000 | **1.000** | 0.000 | Y |
| Podophyllotoxin | 0.000 | 0.000 | **1.000** | 0.000 | Y |
| Vindesine | 0.000 | 0.000 | **1.000** | 0.000 | Y |
| Vinorelbine | 0.000 | 0.000 | **0.999** | 0.000 | Y |
| 5-Fluorouracil | 0.000 | 0.002 | 0.000 | **0.998** | Y |

[a]Probability of the pattern belonging to each output (mechanistic) category. Each prediction is made for a pattern never 'seen' by the network. Topo II, topoisomerase II inhibitors; AA, alkylating agents; TU, tubuline active agents; AM, anti-metabolites; 4-HC, 4-hydroperoxy-cyclophosphamide. Winning neurons are depicted in bold. [b]The chosen smoothing factor of 0.3 provided no detectable output weights for AraC. Increasing the smoothing factor to 0.4 showed a high probability (0.999) for the AA category.

reason for this paradoxical sensitivity remains to be elucidated. Despite this, all tubulin-active drugs were found among the ten drugs with the highest correlation coefficients when correlations to taxol were examined and ranked. Thus, by combining NN analysis, correlation analysis and visual inspection of the correlation graphs, an initial guess on mechanistic type may be made with some confidence, which can help direct further research. However, this approach will require prospective confirmation on additional sets of drugs.

In addition to the mechanistic classification by comparison of drug-specific patterns of drug activity, the present cell line panel can also provide information on the susceptibility of drugs to defined mechanisms of resistance at the molecular level. The calculation of simple ratios of resistant over parental cell line $IC_{50}$ values, 'resistance factors', may provide this complementary information. Although, the mechanisms of resistance have been established using tumour cell lines they may be of clinical importance. The present system may also provide a tool for structure–activity relationship (SAR) studies by giving a simultaneous comparison of quantitative (i.e. relative potency) and qualitative (i.e. presumed mechanism of action) features among a selected group of compounds (Boyd and Paull, 1995). This approach requires no prior knowledge of the structure of the molecular target *per se*. From a practical point of view, using the method described, 6–9 drugs can easily be prepared and analysed on all ten cell lines each week by a single technician. This relatively limited expenditure of resources and workload may also allow single institutions to acquire this capacity.

In the present study we employed PNN instead of classical back propagation networks as used by Weinstein *et al.* (1992). The backward propagation type is highly complex and involves many small modifications of the system parameters that gradually improve system performance. The major difference between PNN and backward propagation is that the sigmoid activation function of the latter is replaced by a statistically derived one in the former (Specht, 1990). The main operational advantage of PNN is that training is very easy and quick (instantaneous) and sparse data are adequate for network performance. The shape of the decision surface can be made as complex as necessary, or as simple as desired, by choosing appropriate values of only one parameter (the smoothing factor; Specht, 1990).

Discovery of new molecular targets and/or mechanisms of resistance is one of the major objectives of the present research programme. This can to some extent be accomplished by using the drug database as described. However, development of a complementary database of differential molecular expression across the cell line panel may add to this objective by providing molecular correlates to the drug activity patterns. By seeding the drug database with across-cell line quantitative patterns of molecular expression/function of cell growth-regulatory and/or drug sensitivity or resistance determinants (in the mean-graph format), matching drug activity patterns can be identified and provide clues on the nature of the particular drug–target interaction. Indeed, the NCI reported very promising results when their drug database was searched using P-gp expression (Alvarez *et al.*, 1995) and function (Lee *et al.*, 1994) as the seed patterns. High correlations of measured across-panel patterns of P-gp function to drug–response patterns of known P-gp substrates have also been observed using the present system (not yet published).

The above approach might provide important information on the molecular pharmacology of drug interactions with *known* target molecules. At a future stage the possibility of developing a database on differential expression of *unknown* molecules using quantitative protein gel electrophoresis

(Anderson *et al.*, 1991) or detection of differential mRNA expression using the differential display polymerase chain reaction (PCR) technique (Liang and Pardee, 1992) might also be explored. Although, the feasibility and utility of such an approach is hard to predict, research in this direction has already begun at the NCI (Weinstein, *et al.*, 1994).

The limited number of cell lines used in the present panel does not allow tumour type specificity of drugs to be evaluated. However, cell lines may not be an optimal model for this purpose. Indeed, tumour type-specific detection of standard drugs in the NCI operated *in vitro* screen has not been convincingly demonstrated (Weisenthal, 1992). In contrast, application of non-clonogenic cell culture assays of primary cultures of tumour cells from patients has been shown to mimic the known clinical activity pattern of standard drugs. We have previously shown that the FMCA can detect tumour type-specific activity retrospectively for a series of standard drugs (Nygren *et al.*, 1994) and prospectively for early phase I–II drugs such as CdA, gemcitabine and taxol (Larsson *et al.*, 1994; Csoka *et al.*, 1995; Nygren *et al.*, 1995). Thus, the parallel or sequential application of these model systems may provide important complementary information on sensitivity, selectivity and similarity of anti-cancer drug action.

Some additional potential limitations should also be considered. The limited number of cell lines (and thus data points) may render the correlation analysis sensitive to errors as one deviating point can have a large impact on the calculated correlation coefficients. This may to some extent be avoided by visual inspection of correlation graphs and recalculation of correlation coefficients after leaving out suspect data points as well as parallel comparison with NN analysis. NN may be less sensitive to single point errors or missing data as the information is encoded throughout the net structure and is relatively less dependent on single data points (Weinstein *et al.*, 1992). In the present study the predictive accuracy of the NN was reasonably good when adhering to the quality criteria established for successful assays.

Furthermore, with the present approach, only drugs with a cytotoxic mode of drug action are readily amenable to analysis due to the relatively short assay time. Thus, compounds with strictly antiproliferative effects might be missed. Partial remedy for this may be to use $IC_{30}$ values, which were used for three of the drugs in the present study, or increased assay time. Increasing assay time will increase the impact of antiproliferative effects especially for slowly proliferating cell lines (Larsson and Nygren, 1989). However, for the anti-metabolites, for which time-dependent antiproliferative effects may be a characteristic, all these drugs produced an $IC_{50}$ value in at least four cell lines.

Finally, for many drugs the mechanism of action is only tentatively defined and may be different and mixed depending on the cell system used. Owing to the limited number of drugs and drug classes tested in the present study, only four mechanistic categories were selected as output categories in the analysis. However, despite these potential limitations the patterns of these four groups at least were fairly well separated to allow good predictions in the majority of cases also when the fifth category for unknown or other mechanism of action was added. Differentiation of these mechanistic categories into subgroups may be possible as the database expands and new knowledge on mechanisms of action is added. Additional cell lines with novel mechanisms of resistance may also be added to the panel in the future.

In summary, new anti-cancer drugs with improved efficacy and a broader spectrum of activity are desperately needed. The present evaluation system may provide important initial information not only on anti-tumor efficacy and mechanistic classification of each drug, but, also on the susceptibility to defined mechanisms of resistance at the molecular level. Moreover, this *in vitro* system may also serve as a practical tool for bioassay-guided drug design. However, further prospective evaluation is required to assess the utility of these potential applications.

**Acknowledgements**
This study was supported by grants from the Swedish Cancer Society and the Lions Cancer Foundation. The skillful technical assistance of Ms Charlotta Sandberg and Mrs Carina Alvfors is gratefully acknowledged.

### References

ALLEY MC, SCUDIERO DA, MONKS A, HURSEY ML, CZERWINSKI MJ, FINE DL, ABBOTT DL, MAYO GH, SHOEMAKER RH AND BOYD MR. (1988). Feasibility of drug screening with panels of human tumor cell lines using a microculture tetrazolium assay. *Cancer Res.*, **48,** 589–601.

ANDERSON LA, ESQUER-BLASKO R, HOFMAN JP AND ANDERSON NG. (1991). A two-dimensional gel data-base of rat liver proteins useful in gene regulation and drug effect studies. *Electrophoresis*, **12,** 907–912.

ALVAREZ M, PAULL A, MONKS A, HOSE C, LEE JS, WEINSTEIN J, GREVER M, BATES S AND FOJO T. (1995). Generation of a drug resistance profile by quantitation of mdr-1/P-glycoprotein in the cell lines of the National Cancer Institute anticancer drug screen. *J. Clin. Invest.*, **95,** 2205–2214.

BAAS F, JONGSMA A, BROXTERMAN H, ARCECI R, HOUSMAN D, SCHEFFER, GL, RIEHORST A, VAN GROENIGEN M, VAN NIEWINT AWM AND JOENJE H. (1990). Non-P-glycoprotein mediated mechanism for multidrug resistance precedes P-glycoprotein expression during in vitro selection for doxorubicin resistance in a human lung cancer cell line. *Cancer Res.*, **50,** 5392.

BECK WT. (1987). The cell biology of multiple drug resistance. *Biochem. Pharamacol.*, **36,** 2879–2887.

BECK WT, CIRTAIN MC, DANKS MK, FELSTED RL, SAFA AR, WOLVERTON JS, SUTTLE DP AND TRENT, JM. (1987). Pharmacological, molecular, and cytogenetic analysis of 'atypical' multidrug-resistant human leukemic cells. *Cancer Res.*, **47,** 5455–5460.

BELLAMY WT, DALTON WS, GLEASON MC, GROGAN TM AND TRENT JM. (1991). Development and characterisation of a melphalan-resistant human multiple myeloma cell line. *Cancer Res.*, **51,** 995–1002.

BOTLING J, LIMINGA G, LARSSON R, NYGREN P AND NILSSON K. (1994). Development of vincristine resistance and increased sensitivity to cyclosporin A and verapamil in the human U-937 lymphoma cell line without over expression of the 170 KDa P-glycoprotein. *Int. J. Cancer*, **58,** 269–274.

BOYD MR. (1993). The future of new drug development. In *Current Therapy in Oncology*, JE Neiderhuber (ed.) pp. 11–22. B.C. Decker: Philadelphia.

BOYD MR AND PAULL KD. (1995). Some practical considerations and applications of the National Cancer Institute in vitro anticancer drug discovery screen. *Drug Dev. Res.*, **34,** 91–109.

COLE S, BHARDWAJ G, GERLACH JH, ALMQUIST KC AND DEELAY RG. (1992). A novel ATP binding casette transporter gene overexpressed in multidrug resistant human lung tumor cells. *Science*, **268,** 1650–1654.

COLE SP, SPARKS KE, FRASER K, LOE DW, GRANT CE, WILSON GM AND DEELEY RG. (1994). Pharmacological characterization of multidrug resistant MRP-transfected human tumor cells. *Cancer Res.*, **54,** 5902–5910.

CSOKA K, LILIEMARK J AND NYGREN P. (1995). Evaluation of the cytotoxic activity of Gemcitabine in primary cultures of tumor cells from patients with hematologic or solid tumors. *Semin. Oncol.*, **22,** 47–53.

DALTON WS, DURIE BG, ALBERTS DS, GERLACH JH AND CRESS AE. (1986). Characterization of a new drug-resistant human myeloma cell line that expresses P-glycoprotein. *Cancer Res.*, **46,** 5125–5130.

DALTON WS, GROGAN TM, RYBSKI JA, SCHEPER RJ, RICHTER L, KAILEY J, BROXTERMAN HJ, PINEDO HM AND SALMON S. (1989). Immunohistochemical detection and quantification of P-glycoprotein in multiple drug-resistant human myeloma cells: association with level of drug resistance and drug accumulation. *Blood*, **73**, 747-752.

DALTON WS, GROGAN TM, MELTZER PS, SCHEPER RJ, DURIE BGM, TAYLOR CW, MILLER TP AND SALMON SE. (1989). Drug-resistance in multiple myeloma and non-Hodgkin's lymphoma: detection of P-glycoprotein and potential circumvention by addition of verapamil to chemotherapy. *J. Clin. Oncol.*, **7**, 415.

DANKS MK, YALOWICH JC AND BECK WT. (1987). Atypical multiple drug resistance in a human leukemic cell line selected for resistance to teniposode (VM-26). *Cancer Res.*, **47**, 1297-1301.

DANKS MK, SCHMIDT CA, CIRTAIN MC, SUTTLE DP AND BECK WT. (1988). Altered catalytic activity of and DNA cleavage by DNA topoisomerase II from human leukemic cells selected for resistance to VM-26. *Biochemistry*, **27**, 8861-8869.

DOYLE LA, ROSS DD, ORDONEZ JV, YANG W, GAO Y, TONG Y, BELANI CP AND GUTHEIL JC. (1995). An etoposide-resistant lung cancer subline overexpresses the multidrug resistance-associated protein. *Br. J. Cancer.*, **72**, 535-542.

GOLDIN A, JM V, MACDONALD J, MUGGIA F, HENNEY J AND DeVITA V. (1981). Current results of the screening program at the Division of Cancer Treatment, National Cancer Institute. *Eur. J. Cancer*, **17**, 129-1 42.

GRINDLEY G. (1990). Current status of cancer drug development: Failure or limited success? *Cancer Cells*, **2**, 163-171.

HALL A AND CATTAN A. (1991). Drug resistance mechanisms in leukemia. *Bailliere's Clin. Hematol.*, **4**, 655-677.

HOCHHAUSER D AND HARRIS A. (1991). Drug resistance. *Br. Med Bull.*, **47**, 178-196.

JENSEN PB, JENSEN PS, DEMANT EJF, FRICHE E, SORENSEN BS, SEHSTEDT M., WASSERMAN K, VINDELOV L, WESTERGAARD O AND HANSEN HH. (1991). Antagonistic effect of aclarubic in on daunorubicin induced cytotoxicity in human small cell lung cancer cells: relationship to DNA integrity and topoisomerase II. *Cancer Res.*, **51**, 5093-5099.

KRAMER R, ZAKHER J AND KIM G. (1988). Role of the glutathione redox cycle in acquired and de novo multidrug resistance. *Science*, **241**, 694.

LARSSON R AND NYGREN P. (1989). A rapid fluorometric method for semiautomated determination of cytotoxicity and cellular proliferation of human tumor cell lines in microculture. *Anticancer Res.*, **9**, 1111-1120.

LARSSON R AND NYGREN P. (1990). Pharmacological modification of multi-drug resistance (MDR) in vitro detected by a novel fluorometric microculture cytotoxicity assay. Reversal of resistance and selective cytotoxic actions of cyclosporin A and verapamil on MDR leukemia T-cells. *Int. J. Cancer*, **46**, 67-72.

LARSSON R, NYGREN P, EKBERG M AND SLATER L. (1990). Chemotherapeutic drug sensitivity testing of human leukemia cells in vitro using a semiautomated fluorometric assay. *Leukemia*, **4**, 567-571.

LARSSON R, KRISTENSEN J, SANDBERG C AND NYGREN P. (1992). Laboratory determination of chemotherapeutic drug resistance in tumor cells from patients with leukemia using a fluorometric microculture cytotoxicity assay (FMCA). *Int. J. Cancer*, **50**, 177-185.

LARSSON R, FRIDBORG H, LILIEMARK J, CSOKA K, KRISTENSEN J, DE LA TORRE M AND NYGREN P. (1994). In vitro activity of 2-chlorodeoxyadenosine (CdA) in primary cultures of human hematological and solid tumors. *Eur. J. Cancer*, **30A**, 1022-1026.

LEE JS, PAULL K, ALVAREZ M, HOSE C, MONKS M, GREVER M, FOJO AT AND BATES SE. (1994). Rhodamine efflux patterns predict P-glycoprotein substrates in the National Cancer Institute drug screen. *Mol. Pharmacol.*, **46**, 627-638.

LIANG P AND PARDEE AB. (1992). Differential display of eukaryotic messenger RNA by means of the polymerase chain reaction. *Science*, **257**, 967-971.

MARSONI S, HOTH D, SIMON R, LEYLAND-JONES B, ROSA De M AND WITTES RE. (1987). Clinical drug development: An analysis of phase 2 trials, 1970-1985. *Cancer Treat. Rep.*, **71**, 71-80.

MIRSKI SE, GERLACH JH AND COLE SP. (1987). Multidrug resistance in a human small cell cancer cell line selected in adriamycin. *Cancer Res.*, **47**, 2594-2598.

MONKS A, SCUDIERO D, SKEHAN P, SHOEMAKER R, PAULL K, VISTICA D, HOSE C, LANGLEY J, CRONISE P, VAIGRO-WOLFF A, GRAY-GOODRICH M, CAMBELL H, MAYO J AND BOYD M. (1991). Feasibility of a high-flux anticancer drug screen using a diverse panel of cultured human tumor cell lines. *J. Natl Cancer Inst.*, **83**, 757-765.

MULCAHY RT, BAILEY HH AND GIPP JJ. (1994). Up-regulation of gamma-glutamylcyateine synthetase activity in melphalan-resistant human multiple myeloma cells expressing increased glutathione levels. *Cancer Chemother. Pharmacol.*, **34**, 67-71.

NAGOURNEY RA, EVANS SS, MESSENGER JC, ZHUANG SU Y AND WEISENTHAL LM. (1993). 2 chlorodeoxyadenosine activity and cross resistance patterns in primary cultures of human hematologic neoplasms. *Br. J. Cancer*, **67**, 10-14.

NYGREN P AND LARSSON R. (1990). Verapamil and cyclosporin A sensitize human kidney tumour cells to vincristine in absence of membrane P-glycoprotein and without apparent changes in the cytoplasmic free $Ca^{2+}$ concentration. *Biosci. Rep.*, **10**, 231-237.

NYGREN P AND LARSSON R. (1991). Differential in vitro sensitivity of human tumor and normal cells to chemotherapeutic agents and resistance modulators. *Int. J. Cancer*, **48**, 598-604.

NYGREN P, KRISTENSEN J, SUNDSTRÖM C, LÖNNERHOLM G, KREUGER A AND LARSSON R. (1992). Feasibility of the fluorometric microculture cytotoxicity assay (FMCA) for cytotoxic drug sensitivity testing of tumor cells from patients with acute lymphoblastic leukemia. *Leukemia*, **6**, 1121-1128.

NYGREN P, FRIDBORG H, CSOKA K, SUNDSTRÖM C, de la TORRE M, KRISTENSEN J, BERGH J, HAGBERG H, GLIMELIUS B, RASTAD J, THOLANDER B. AND LARSSON, R. (1994). Detection of tumor-specific cytotoxic drug activity in vitro using the fluorometric microculture cytotoxicity assay and primary cultures of Tumor cells from patients. *Int. J. Cancer*, **56**, 715-720.

NYGREN P, CSOKA K, JONSSON B, FRIDBORG H, BERGH J, HAGBERG H, GLIMELIUS B, BRODIN O, THOLANDER B, KREUGER A, LÖNNERHOLM G, JAKOBSSON Å, OLSEN L, KRISTENSEN J AND LARSSON R. (1995). The cytotoxic activity of Taxol in primary cultures of tumor cells from patients is partly mediated by Cremophore EL. *Br. J. Cancer*, **71**, 478-481.

OHTA S, NISHITO K, KUBO S, NISHIO M, OHMORI T, TAKAHASHI T AND SAIJO N. (1993). Characterisation of a vindesine-resistant human small-cell lung cancer cell line. *Br. J. Cancer*, **68**, 74-79.

PAULL KD, SHOEMAKER RH, HODES L, MONKS A, SCUDIERO DA, RUBINSTEIN L., PLOWMAN J AND BOYD MR. (1989). Display and analysis of patterns of differential activity of drugs against human tumor cell lines: Development of mean graph and COMPARE alogrithm. *J. Natl Cancer Inst.*, **81**, 1088-1092.

SLOVAK ML, HO JP, BHARDWAJ G, KURZ EU, DEELEY RG AND COLE SP. (1993). Localization of a novel multidrug resistance-associated gene in the HT1080/DR4 and H69AR human tumor cell lines. *Cancer Res.*, **53**, 3221-3225.

SPECHT DF. (1990). Probabilistic neural networks. *Neural Networks*, **3**, 109-118.

VAN KALKEN C, PINEDO H AND GIACCONE G. (1991). Multidrug resistance from a clinical point of view. *Eur. J. Cancer*, **27**, 1481-1486.

VAN OSDOL WW, MYERS TG, PAULL KD, KOHN KW AND WEINSTEIN J. (1994). Use of the Kohnen self-organization map to study the mechanisms of action of chemotherapeutic agents. *J. Natl Cancer Inst.*, **86**, 1853-1859.

WEINSTEIN JN, KOHN KW, GREVER MR, VISVANADHAN VN, RUBINSTEIN LV, MONKS AP, SCUDIERO DA, WELCH L, KOUTSOUKOS AD, CHIAUSA AJ AND PAULL KD. (1992). Neural computing in cancer drug development: Predicting mechanism of action. *Science*, **258**, 447-451.

WEINSTEIN JN, MYERS T, BUOLAMWINI J, RAGHAVAN K, van OSDOL WW, LICHT J, VISWANADHAN VN, KOHN KW, RUBINSTEIN LV, KOUTSOUKUS AD, MONKS A, SCUDIERO DA, ANDERSON NL, ZAHAREVITZ D, CHABNER BA, GREVER MR AND PAULL KD. (1994). Predictive statistics and artificial intelligence in the US National Cancer Institute's drug discovery program for cancer and AIDS. *Stem Cells*, **12**, 13-22.

WEISENTHAL LM. (1992). Antineoplastic drug screening belongs in the laboratory, not in the clinic. *J. Natl Cancer Inst.*, **84**, 466-469.